# 3D Stent Reconstruction Using CMCT: A Novel Method to Assess Stent Expansion

**DOI:** 10.1016/j.jaccas.2024.102248

**Published:** 2024-02-09

**Authors:** Hachem-Ali Haidar, Matthieu Perier, Hakim Benamer

**Affiliations:** a Department of Interventional Cardiology, Hopital Foch, Suresnes, France; b Department of Interventional Cardiology, European Hospital of Paris La Roseraie, Aubervilliers, France; c Cardiovascular Institute Paris Sud, Massy, France

**Keywords:** 3-dimensional, C-arm motion compensated computed tomography, stent underexpansion

## Abstract

Stent underexpansion in calcified coronary stenosis is an important predictor of major short- and long-term adverse cardiovascular events. In this case, we describe a novel method for assessing stent expansion using 3-dimensional stent reconstruction with C-arm motion compensated computed tomography.

## Graphical abstract

**Figure:**
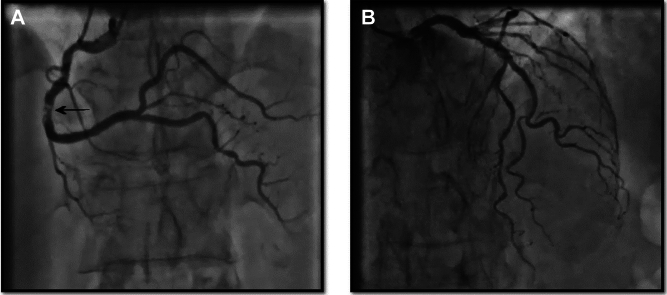

## History of Presentation

The patient was a 71-year-old man who was referred to our center for coronary angiography because of cardiac angina (Canadian Cardiovascular Society III). Two weeks before admission, he reported sudden onset acute retrosternal chest pain. He was diagnosed with an inferior wall myocardial infarction that was medically managed with dual antiplatelet therapy and anticoagulation. He did not undergo coronary angiography at the time and was discharged on aspirin 100 mg, clopidogrel 75 mg, atorvastatin 40 mg, atenolol 100 mg, and ramipril 5 mg. He had an unremarkable physical examination, and his vital signs were within the normal range.

**Learning Objectives**

• To recognize the importance of intravascular imaging and plaque modification techniques in the setting of severe calcified lesions to decrease short- and long-term complications.
• To evaluate a novel method to assess stent expansion with 3D stent visualization using CMCT and compare it to optical frequency domain imaging.

## Past Medical History

The patient had no significant medical history. Prior smoking and his age were cardiovascular risk factors.

## Differential Diagnosis

The differential diagnosis included stable angina, microvascular angina, and noncardiac chest pain.

## Investigations

Electrocardiography showed Q waves in the inferior leads. A chest X-ray was unremarkable. Transthoracic echocardiography showed akinesis of the inferior wall with a preserved ejection fraction around 55%. There was nonsignificant valvular disease. His blood work was unremarkable; notably, his troponin T was 5 ng/mL (normal <0.14 ng/mL).

## Management

**Figure 1:**
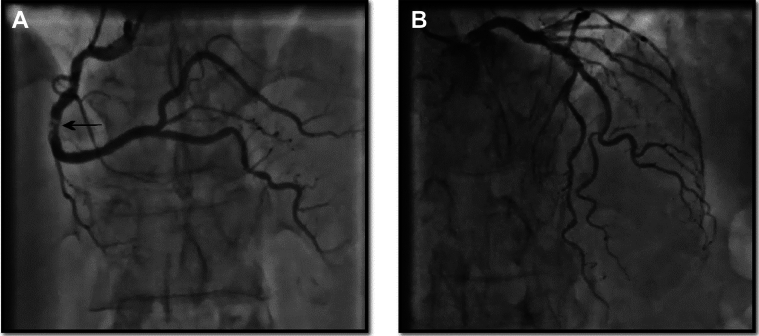
Right and Left Coronary Artery Angiograms (A) Right coronary artery (RCA) in the right anterior oblique (RAO) 30°, cranial 15° view showing a severe calcified stenosis in the mid-RCA (arrow). (B) Left coronary artery in the RAO 12°, cranial 37° view showing nonsignificant atheroma.

We proceeded to perform coronary angiography given the patient’s clinical presentation. It showed severe calcified stenosis in the mid–right coronary artery (RCA) with TIMI flow grade 3 (Figure 1). Because of inferior wall akinesis and the Q-wave in the inferior leads, we performed single-photon emission computed tomography with injection of thallium-201, which showed inferior wall viability. Given the persistence of angina symptoms and the viability of the inferior wall, we decided to proceed with angioplasty.

**Figure 2:**
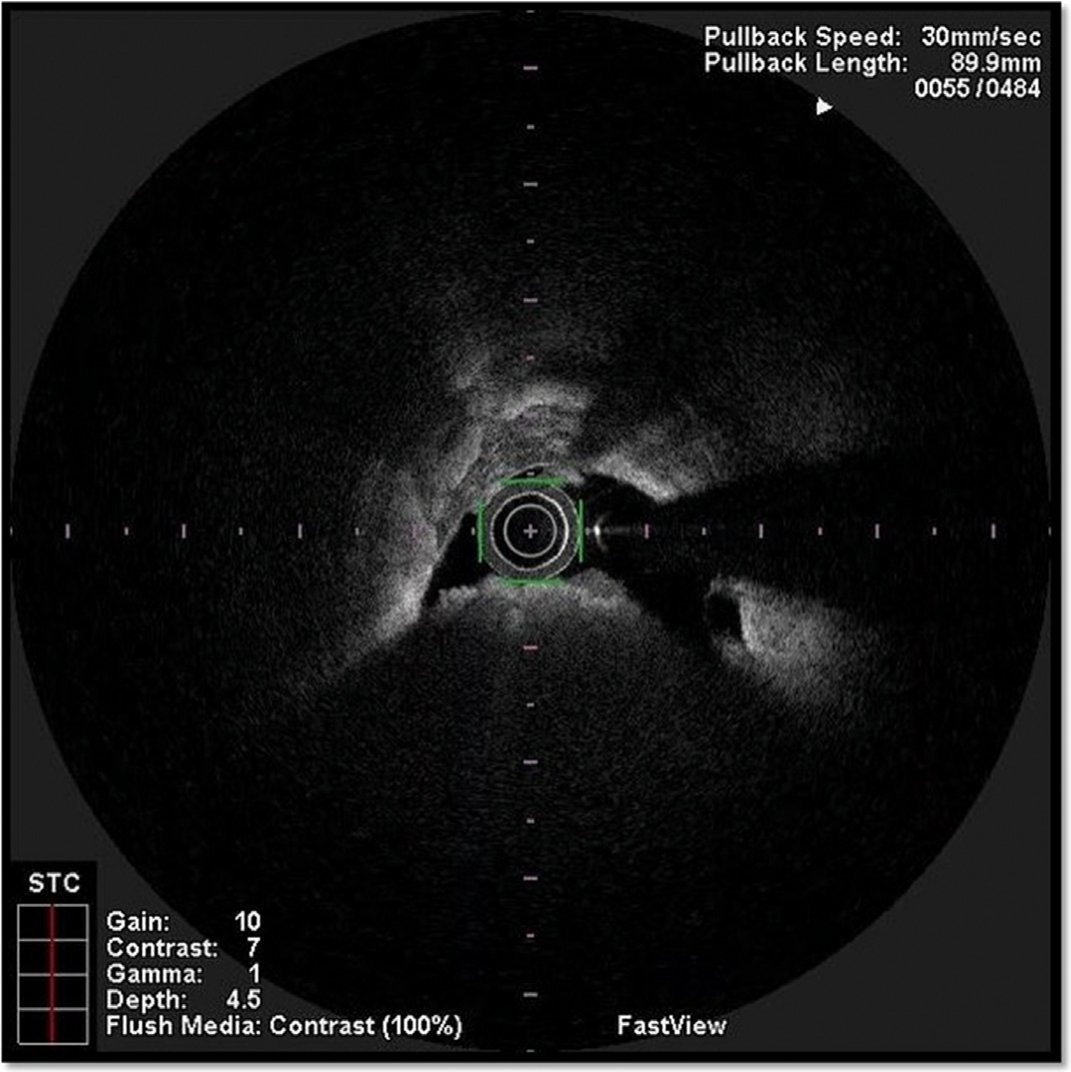
Optical Frequency Domain Imaging Pull Back of the RCA Severe stenosis of the right coronary artery (RCA) with a calcified nodule and concentric calcifications (arc >180°).

Balloon predilation was performed with a 2.5 × 12 mm compliant balloon followed by intravascular imaging with optical coherence tomography using the Lunawave optical frequency domain imaging (OFDI) device (Terumo). The OFDI pull back showed severely calcified stenosis in the mid-RCA (Figure 2, Video 1).

**Figure 3:**
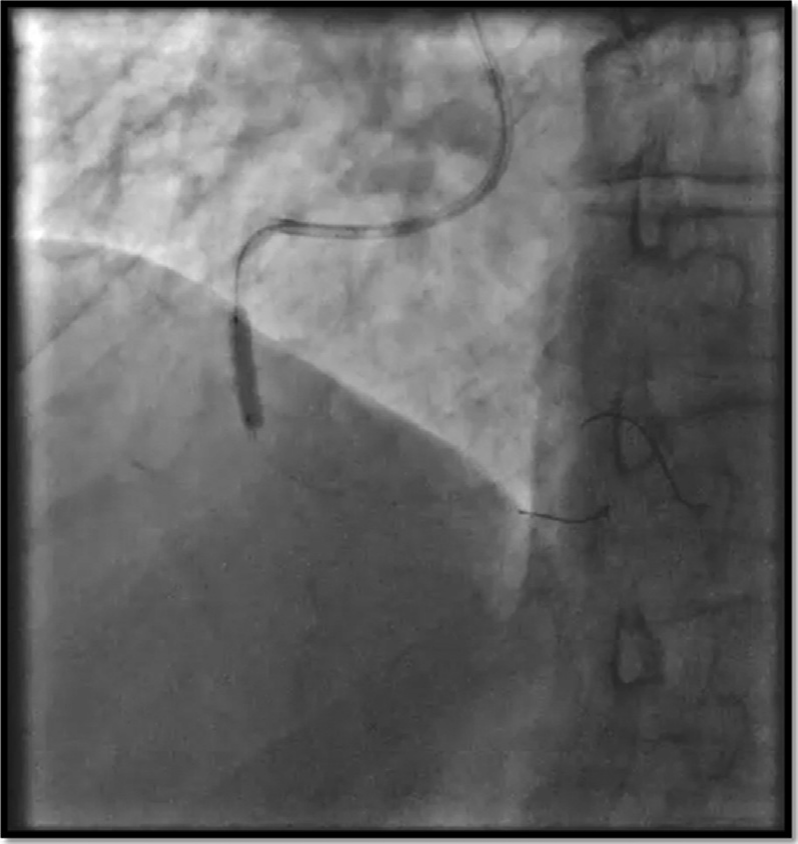
Balloon Predilation After Rotational Atherectomy Good balloon (3.5 mm × 15 mm OPN NC) expansion is noted.

We performed 3 runs of rotational atherectomy using the 1.50-mm ROTALink Plus Burr (Boston Scientific) followed by balloon dilation with a 3.5 × 15 mm OPN NC (SIS-medical) balloon, which expanded nicely as shown in angiography (Figure 3). We deployed 2 drug-eluting stents ULTIMASTER TANSEI [Terumo] 4.0 × 24 mm in the mid-RCA and 4.0 × 28 mm in the proximal RCA) with good angiographic results.

**Figure 4:**
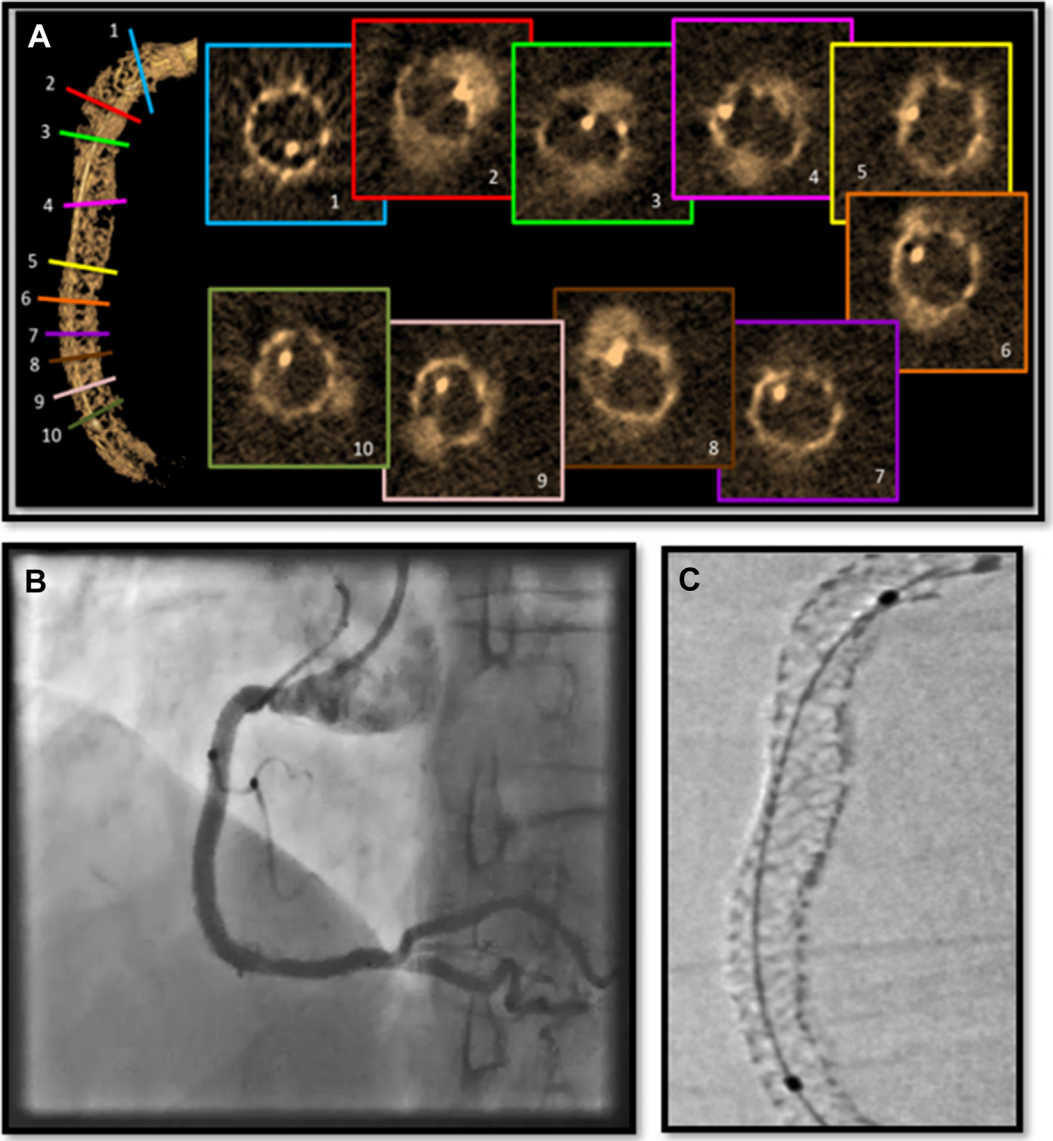
3D Stent Visualization, Stent Enhancement, and Final Angiographic Result (A) Three-dimensional (3D) stent visualization with multislice images (3DStent prototype). (B) The final angiographic result. (C) Classic fluoroscopic cine 2-dimensional stent enhancement (StentViz).

At the end, we performed classical stent enhancement visualization (Stent Viz, GE HealthCare) and 3-dimensional (3D) visualization of the stent with a prototype of the 3DStent (GE HealthCare) (Figure 4, Video 2) before performing an OFDI pull back that showed a distal edge dissection that was treated by implanting a third drug-eluting stent in the proximal segment of the distal RCA. Stent expansion was adequate, and no significant malapposition was noted on the final OFDI pull back (Video 3).

## Discussion

Stent underexpansion, especially in the setting of calcified lesions, is a major risk factor for early and late stent thrombosis, which can have devastating effects and life-threatening consequences.^1^ The use of adequate plaque modification techniques, such as intravascular lithotripsy and rotational, orbital, or laser excimer atherectomy, especially in the presence of severely calcified lesions, is of the utmost importance to ensure proper stent expansion^2^ and reduce early and late onset complications. Intravascular imaging techniques and/or stent enhancement techniques after stent deployment are usually used to assess proper stent expansion. There is considerable variability in the published literature regarding the definition of underexpansion.^3^ The European Association of Percutaneous Cardiovascular Interventions expert consensus document suggests a minimal stent area <80% of the average reference area ratio cutoff to define clinically significant underexpansion.^4^

However, because of financial constraints and regulatory and reimbursement issues,^5^ intravascular imaging techniques are not widely used even in high-income countries.^6^ Classical digital stent enhancement can detect stent underexpansion,^7^ but because it can only provide a 2-dimensional view (Figure 4C), its accuracy remains limited compared to intravascular imaging.

In this context, 3D visualization of the stent after its deployment using the 3DStent C-arm motion compensated computed tomography (CMCT) technology is a new technique that improves the detection of stent underexpansion compared to classical digital stent enhancement; 3D stent reconstruction relies on rotational cine acquisition around the stent from the right anterior oblique 100° to the left anterior oblique 100° (Video 4). The acquisition workflow is largely automated. The operator must center the stent in 2 angulations and verify during an X-ray–free test that the rotation is collision free. Once this is done, rotational cine acquisition can be easily performed with an automatic synchronization of X-ray exposure and gantry rotation. During the acquisition, like for digital stent enhancement, a deflated balloon must be kept inside the stent.

Rotational cine acquisition (ie, rotational fluoroscopy) can already be performed in most of the catheterization laboratories using their already installed image-guided therapy systems and has been used for many years with static anatomies. GE HealthCare’s CMCT 3DStent technology allows the scope of rotational fluoroscopy to be extended to moving coronary stents thanks to ad hoc software processing designed to handle the motion.

**Figure 5:**
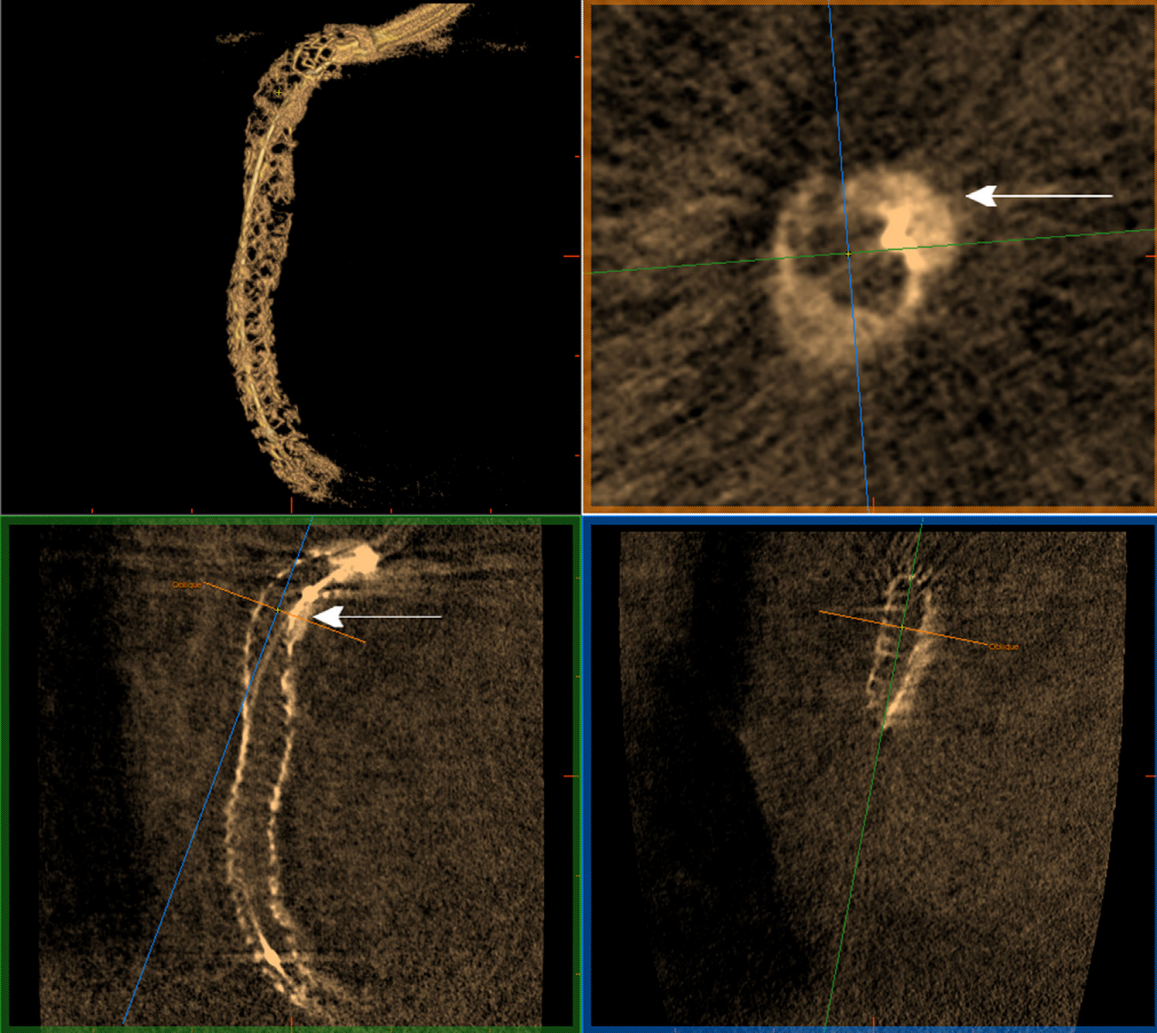
3D Stent and MPR 3D stent visualization and multiplanar reconstruction (MPR) showing severe calcifications in the proximal segment of the RCA with good stent expansion (arrow). Abbreviations as in Figures 1 and 4.

The software uses the acquired frames to automatically generate a 3D model and allows for multiplanar reconstruction that facilitates the evaluation of the stent architecture (Figure 5). It also permits the evaluation of the area across the stent’s length by generating multislice images, thus allowing a more precise assessment of the stent’s expansion.

The main drawback remains, as in all digital stent enhancement techniques, the increased radiation exposure and the inability to detect other stent-related features such as malapposition. On the other hand, it represents a quick, safe, and easy-to-use technique that can be helpful in the catheterization laboratory, especially in the absence of intravascular imaging techniques.

**Figure 6:**
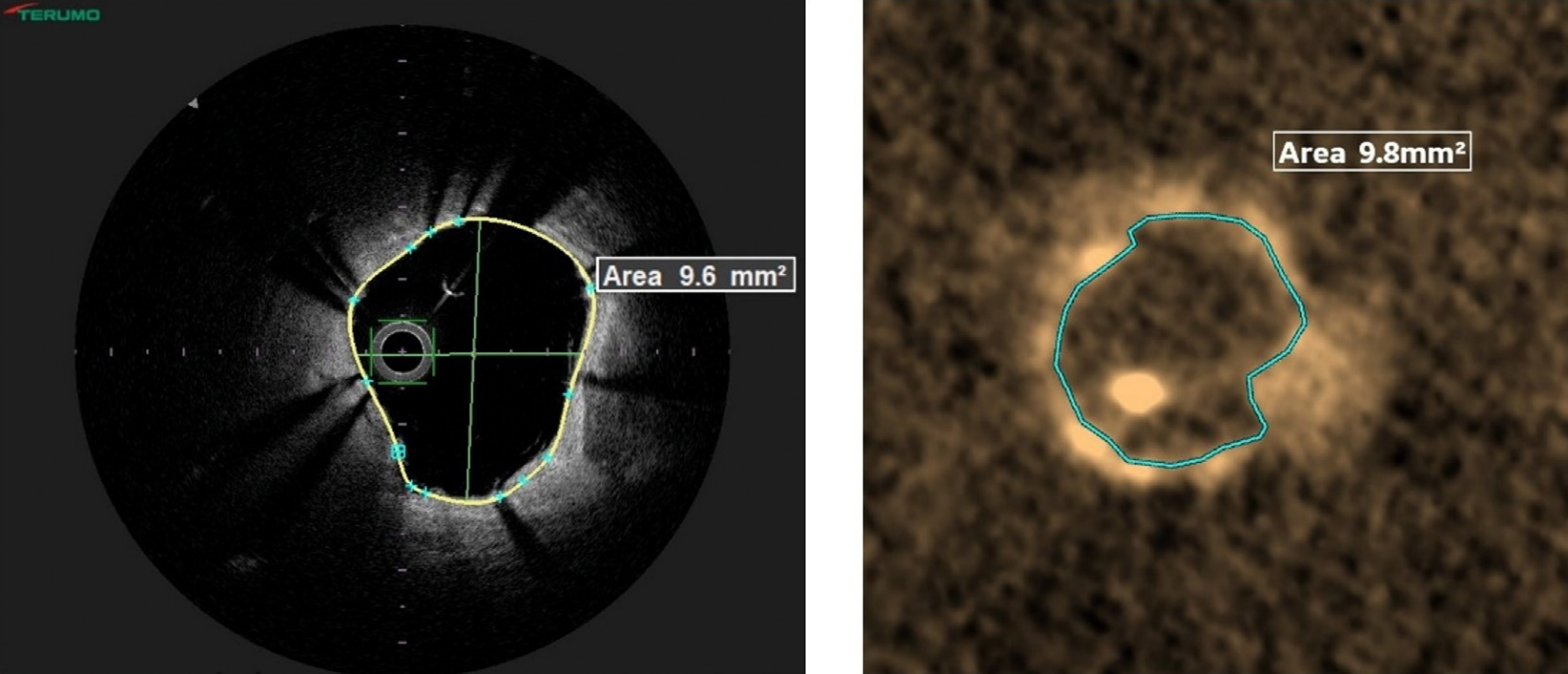
Minimal Stent Area The minimal stent area measured by optical frequency domain imaging and 3-dimensional stent visualization.

In our case, the minimal stent area was 9.6 mm^2^ when measured by OFDI and 9.8 mm^2^ when measured by the 3DStent prototype’s multiplanar reconstruction (Figure 6). The minimal stent area/average reference lumen area was >80% in both cases.

## Conclusions

This case study illustrates the importance of using multimodality imaging to ensure adequate deployment of stents, especially in the setting of calcified lesions. In this case, we describe a new technique, 3D stent reconstruction using CMCT, that ensures good stent expansion identification compared with OFDI; however, further investigations and trials are required to validate this finding.

## Funding Support and Author Disclosures

This work was funded by GE HealthCare. The authors have reported that they have no relationships relevant to the contents of this paper to disclose.

## Appendix

Video 1 Initial OFDI Pull-Back

Video 2 3D Stent Reconstruction

Video 3 Final OFDI Pull-Back

Video 4 3D Stent Cine Acquisition
